# A review of emerging trends in nanomaterial-driven AI for biomedical applications

**DOI:** 10.1039/d5na00032g

**Published:** 2025-04-25

**Authors:** Subhendu Chakroborty, Nibedita Nath, Sameeta Sahoo, Bhanu Pratap Singh, Trishna Bal, Karunesh Tiwari, Yosief Kasshun Hailu, Sunita Singh, Pravin Kumar, Chandra Chakraborty

**Affiliations:** a School of Basic Sciences, Department of Chemistry, Chandigarh University Uttar Pradesh Unnao India subhendu.cy@gmail.com; b Department of Chemistry, D. S. Degree College Laida Sambalpur 768214 Odisha India nibeditanath.mami@gmail.com; c Department of Computing & Information Sciences, Chandigarh University Uttar Pradesh Unnao India; d Department of Pharmaceutical Sciences and Technology, Birla Institute of Technology Mesra Ranchi 835215 India trishna.bal@gmail.com; e Department of Physics, Mai Nefhi College of Science, Eritrean Institute of Technology Mai Nefhi Eritrea tdrkarunesh@gmail.com; f IES College of Education, IES University Bhopal Madhya Pradesh 462044 India; g Inter University Accelerator Centre New Delhi 110067 India; h Department of Allied Sciences, Graphic Era (Deemed to be University) Clement Town 248002 Dehradun India

## Abstract

The field of artificial intelligence (AI) is expanding quickly. To mimic the structure and biological evolution of the human brain, AI was developed to enable computers to acquire knowledge and manipulate their surroundings. There have been notable developments in the use of AI in healthcare; it can enhance diagnosis and treatment in various medical specialties. The cost of prompt diagnosis and treatment is hampered by the absence of efficient, dependable, and reasonably priced detection and real-time monitoring. Smart health tracking systems integrating AI and nanoscience are an emerging frontier that solves these obstacles. Targeted delivery of drug systems, biosensing, imaging, and other diagnostic and therapeutic fields can widely benefit abundantly from nanoscience in healthcare. AI technology has the potential to expand biomedical applications by analyzing and interpreting biological data, speeding up drug discovery, and identifying novel molecules with predictive behavior. This review outlines the current obstacles and potential opportunities for delivering personal healthcare using AI-assisted clinical decision support systems.

## Abstract 2

There is a significant chance that the convergence of AI and nanotechnology will affect numerous other scientific domains.

## Graphical abstract

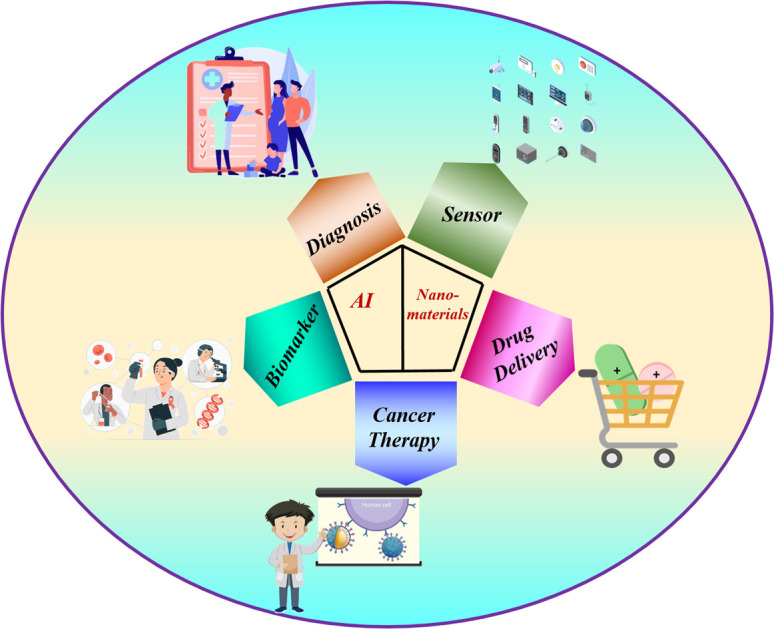

## 1. Introduction

Nanotechnology and AI have received a lot of focus recently and have the capacity to change several facets of our lives substantially. Scientists and researchers are breaking new ground in medical care, technology, energy, and other fields by integrating the unique features of nanotechnology with the power of AI.^1^ The field of computer science that focuses on the creation of intelligent machines that are capable of performing activities that often call for human intelligence is known as artificial intelligence, or simply AI.^2,3^

Scientists can speed up the development of new materials, streamline production procedures, and enhance the functionality of nanoscale devices by incorporating AI approaches into nanotechnology research and manufacturing operations.^4^ The integration of nanotechnology and AI holds significant promise for tackling some of the most pressing problems of society, such as healthcare and environmental sustainability. The combination of AI with nanotechnologies has the potential to significantly contribute to the advancement of several fields, including energy-efficient products, smart environmental monitoring systems, personalized medicine, and early illness diagnosis.^5^

**Fig. 1:**
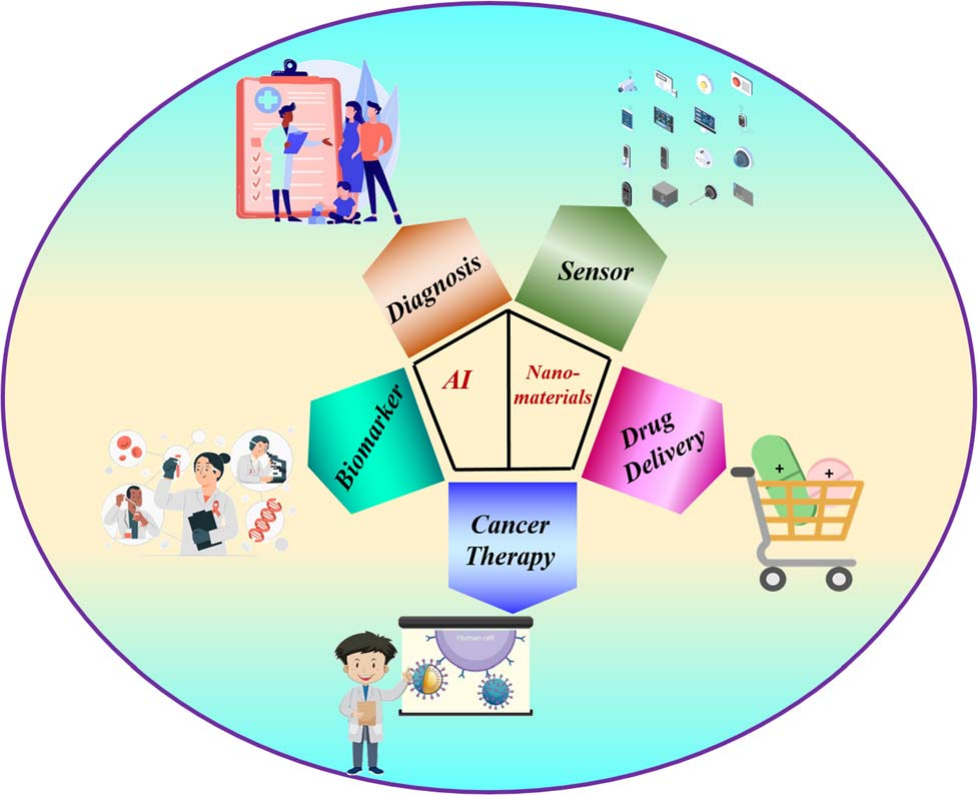
There is a significant chance that the convergence of AI and nanotechnology will affect numerous other scientific domains.

For almost a decade, there has been a prediction that biology, nanotechnology, and AI will come together to create an evolved scientific and technical era. However, efforts to integrate interdisciplinary research are still ongoing. While some of AI's most successful paradigms, such as artificial neural networks and algorithmic evolution, are based on biology, nanotechnology integrates the concepts of chemistry, physics, and engineering. Bridging the gap between currently applicable nanoscience and AI-incorporated nanoscience will help build new communication and technologies with the possibility of biology and technology to coexist that will greatly impact our society (Fig. 1).^6^

The scientific and engineering methods of developing, manufacturing, and evaluating devices and materials with functioning structures on a nanoscale in at least one dimension are collectively referred to as nanotechnology. As it affects the fundamental molecular structure, which in turn affects its chemical and physical properties, it is critical to consider the behavior of individual molecules and groups of interacting molecules regarding the overall properties of the material or devices at these scales.^7^ Nanotechnology is interdisciplinary and has multiple uses in biomolecular detection, therapeutics, microelectronics, DNA sequencing, optics, and sensors. In recent times, it has gained huge importance in the field of pharmaceutical sciences.^8^

The biomedical profession relies heavily on nanomaterials, and with the advent of the digital age, AI has emerged as a useful tool for developing nanomaterials at every stage of the process, from designing to synthesis and characterization. A subset of medical materials created, manufactured, or improved at the nanoscale are known as biomedical nanomaterials.^9,10^ These nanomaterials are useful for cellular illness diagnosis, therapy, and prevention due to their unique biological characteristics and roles.^11–13^ With the ability to precisely regulate a material's structure and properties at the nanoscale, nanomaterials hold enormous potential for use in biological systems.^14,15^

AI and nanotechnology are two innovative fields that have the potential to revolutionize many aspects of daily life, science, and technology.^16^ Controlling and modifying materials on a very small scale, typically 1–100 nm, is the main goal of nanotechnology.^17^ Materials of this size exhibit properties and behaviors distinct from those of larger scales. The development, synthesis, characterization, and application of nanostructures and nanodevices are collectively called nanotechnology, which has applications in various fields, including electronic devices, healthcare, power, and environmental studies. Its scope includes developing novel materials, production techniques, and tools with specialized applications. These solutions address issues in areas such as environmental remediation, generation of renewable energy, and illness detection and treatment. By offering novel approaches to health care,^18,19^ medication delivery,^20^ and diagnostics,^21^ contemporary nanotechnology applications, such as carbon dots for drug research, are transforming healthcare services.^22^ To improve patient results, safety, and the efficiency of medication delivery, much development and research has been conducted on nanomaterials, nanocarriers, & nanostructures. Targeted medicine delivery made possible by nanotechnology minimizes off-target effects and lowers systemic toxicity.

## 2. Artificial intelligence

Building machines that can mimic human intelligence and do tasks that normally require human cognition is the goal of AI, a cutting-edge area of computer science.^23^ With the main goal of replicating and automating cognitive processes such as learning, perception, reasoning, & problem-solving, innovative approaches such as machine learning, computer vision, robotics, neural networks & natural language processing are all included in its wide field.^24^

Machine learning deals with creating techniques that let computers learn from data, analyze them, and improve the outcome over time without specialized programming. Numerous industries, including banking, health care, entertainment, and transportation, have used AI. AI-powered systems in healthcare can estimate disease outcomes, assess medical imaging, and aid in drug discovery. Algorithmic trading, automated customer service, and the detection of fraudulent transactions are all applications of AI algorithms in the financial industry. Autonomous vehicles can currently navigate and make choices in challenging circumstances as a result of the application of AI.^25^

Researchers and biomedical sectors are interested in AI because of its capacity to process vast volumes of data, generate precise results, and regulate processes to achieve the best possible conclusion. AI isn't new as robots are already used for long-term disease prediction and decision-making. In the modern world, technology and algorithms help with the day-to-day chores. Acceptance, transparency, clarity, fairness, and availability are among the major factors considered in accurate machine-algorithm-coordinated results. AI is the capacity of robots or computers to simulate human intelligence using software and algorithms.^26^ Intelligent activities such as knowledge-based learning, assisted surgery, medication discovery, logical reasoning, and advanced imaging can be performed using AI. The concept of AI might seem new, but it was initially created by several scholars in the late 1940s^27–29^, and their work is still useful as a foundation for contemporary research and advancements in AI.

AI-powered systems are also using patient histories and medical records to tailor each person's exact pharmaceutical regimen and optimal course of treatment. Health services can readily receive data about heart rates and activities from wearable health-tracking devices. Because there is a large amount of data from various sources, AI is utilized to analyze the data and discover irregularities for certain individuals. In the same way, data collected by hospitals specific to patients' health monitoring devices might detect possible crises and alert medical professionals. Healthcare system analysis is already being used in some nations, such as Denmark and Norway, to identify inefficiencies in process and treatment errors.^30^ AI-based robots can create novel surgical techniques by analyzing data from prior surgeries. These robots can treat patients more accurately and with fewer accidental movements.^31^ In addition to spine surgery, AI finds use in minimally invasive surgery, robotically assisted procedures, and post-surgical care, including recovery time calculation.^32^

## 3. Basic principles of AI

To aid in future comprehension, the division of AI into learning and nonlearning algorithms is presented. Algorithms in nonlearning systems respond to specific circumstances in a predetermined manner. The system reacts in the same manner every time. Thus, it is incapable of learning. This is how, for instance, classic chess programs are programmed. More complex AI systems, on the other hand, employ a method in which the systems learn. In classical computing, incoming data are fed into an algorithm, also known as a program, which processes them and then returns the output to the user. A pocket calculator is the most basic example in this context. The calculator's actual program remains unchanged while performing a mathematical operation, indicating that the program is not learning. It's not the same as learning AI algorithms.^32^ These algorithms undergo processing changes as they are fed data, indicating that they are learning. The two main categories of learning systems are neural networks that simulate the human brain (deep learning) and other designs. Inspired by the concept of brain architectural replication, deep learning is a subclass of machine learning that uses artificial neural networks (ANNs). Neurons are organized in layers to form the natural brain and dendrites are junctions where these neurons receive inputs. Neurons produce an action potential sent to other neurons in different layers *via* the axon when the sum of the inputs reaches an activation potential. A node is the most fundamental part of an ANN, much like a neuron. Other nodes provide input to this node.^32^ The foundation of deep learning models is ANNs, which comprise interconnected layered nodes that enable hierarchical learning. Data move across these layers during model training, making it possible to extract more intricate features from unprocessed inputs such as text, music, or images.^33^ Over time, several network architectures were created to handle various data and issues. Convolutional neural networks were the next most developed type after fully connected neural networks.^34^ These days, there are also many more complex networks, such as generative adversarial neural networks and U-Nets. Examples of successful applications for these networks include biomarker discovery, prescription prediction, and diagnosis prediction.^35–37^

**Fig. 2:**
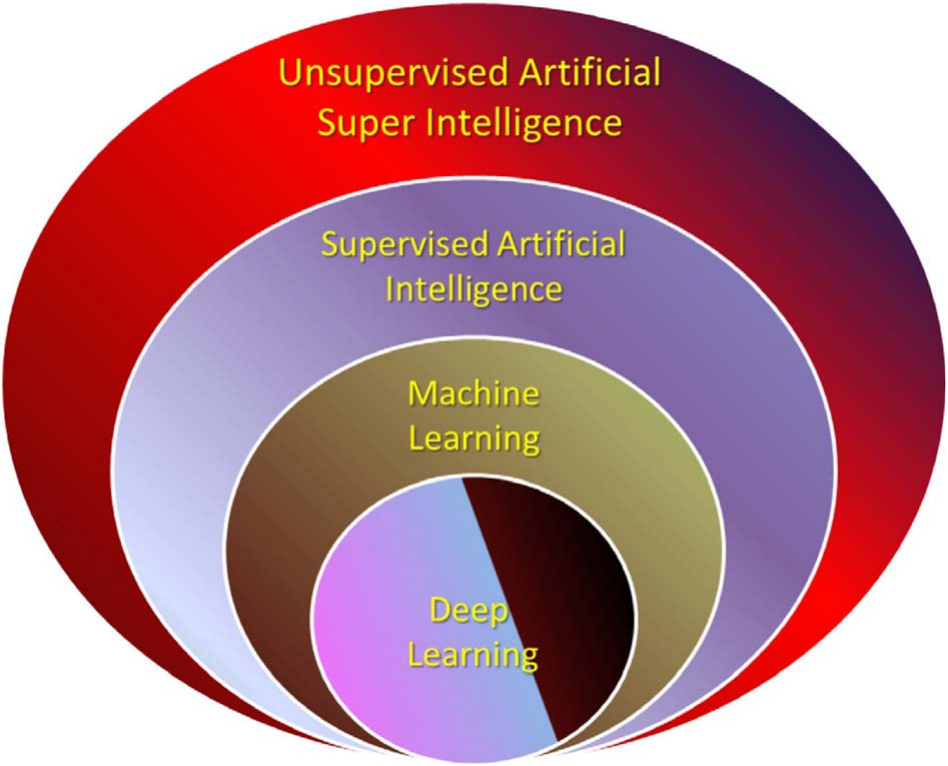
Various levels of AI. Reprinted with permission from ref. 39. Copyright 2020 Nova Science Publishers.

Over the past few decades, artificial intelligence has been on the rise in terms of new technology. It is impossible to overlook the potential and functionality of incorporating this technology with other technologies.^38^ AI augmentation and integration appear to be essential for data mining, analysis of data, and ultimately data prediction whenever we deal with large amounts of data, both structured and unstructured. Data warehousing, in which we have a store of historical data that must be compared to incoming and current data to avoid replicating or duplicating current ones based on their history, is one of the criteria of such analysis. Deep Learning (DL) is one of the AI subsystem parts for this purpose. In DL, incoming data are compared to historical data, and the results are filtered before moving on to Machine Learning (ML), another AI subsystem element. The AI layer is the part that communicates with a person for any fast-paced, real-time, and accurate decision-making process. It is illustrated as the top layer of the integrated system with ML and DL in Fig. 2.^39^ As we can see, the delivery and prescription of drugs at micron and submicron sizes are no exceptions to this role either. When we delve deeply into the world of small things with the size and scale of microns and submicrons, we end up with a lot of data that we gather from it to improve our knowledge and gain the ability to make a correct decision. As a result, we can readily draw the conclusion that, given the data, both historical and current, real-time analysis necessitates AI and human collaboration, as a suitable partner is something that is unavoidable, and, in fact, they unquestionably complement one another.^40^

We can easily see a significant augmentation and integration of mixed AI systems (*i.e.*, AI, ML, and DL) in health and medical science today, where researchers work side by side. Since this integration, which has been seen at the level of nanoscience and nanotechnology, has contributed to the success of these two fields over the past 70 years, we attempt to define and explain AI and its subsets, ML and DL, at the beginning of the chapter. Next, we present the science of nanoengineering and nanotechnology.^41^

## 4. Role of AI in health care

Healthcare costs are skyrocketing. The condition is exacerbated by increasing life expectancy, a sharp increase in the prevalence of long-term illnesses, and the continuous development of expensive new medications. Therefore, it is understandable that scholars have made pessimistic forecasts about the sustainability of medical facilities worldwide. AI offers ways to mitigate the impact of these developments by improving and reducing the expense of healthcare.^42^

AI is increasingly used in healthcare to improve the efficacy and accuracy of healthcare diagnosis, treatment plans, and decision-making.^43^ It might improve patient outcomes and alter the way healthcare is delivered. AI in healthcare has a number of uses.^44^ One method AI is being used in the healthcare sector is by developing machine learning algorithms that can examine vast amounts of patient data and identify patterns and trends that may not be immediately apparent to humans.^45^ This can be especially helpful in spotting early symptoms of illnesses or ailments, enabling an earlier diagnosis, course of treatment, assessment of prognosis, and more. Another application of AI in healthcare is natural language processing (NLP). With NLP systems being able to evaluate electronic medical records (EMRs) and retrieve relevant information, healthcare providers can have easy access to patient data.^46^

Better patient care and more informed decision-making can result from healthcare providers' easier access to and interpretation of patient data. AI can also help with jobs such as image analysis, which enables more precise analysis and effective diagnosis with medical imaging such as X-rays or CT scans.^47^ It is anticipated that AI will be used in healthcare much more frequently.^48^ Examples of contemporary uses include disease diagnosis, personalized medicine, drug research, gene editing, & health care support.^49^

It is impossible to exaggerate the importance of AI applications in healthcare. It is impossible to underestimate the importance of AI applications in healthcare, which have enormous potential to revolutionize real-time health condition monitoring, tailored treatment, disease diagnosis, and operational healthcare management for transforming the fields of disease diagnosis, personalized medicine, real-time health condition monitoring, and operational healthcare management. AI-driven diagnostic technologies, for example, can effectively evaluate medical images and often pick up on details that the human eye might miss. This level of accuracy translates to earlier and more precise diagnoses, dramatically improving patient outcomes. Similarly, AI algorithms are making significant strides toward genuinely personalized medicine in the area of treatment personalization. They can comb through enormous datasets to find patterns and forecast which medicines will work best for particular patient profiles. AI is also used in monitoring patients, where wearable technology and remote monitoring equipment provide ongoing patient health oversight, allowing for prompt treatments and a decrease in readmissions to hospitals. AI has the potential to improve efficiency and patient satisfaction in the delivery of healthcare by streamlining processes such as appointment scheduling and hospital workflow optimization.^50–52^

**Fig. 3:**
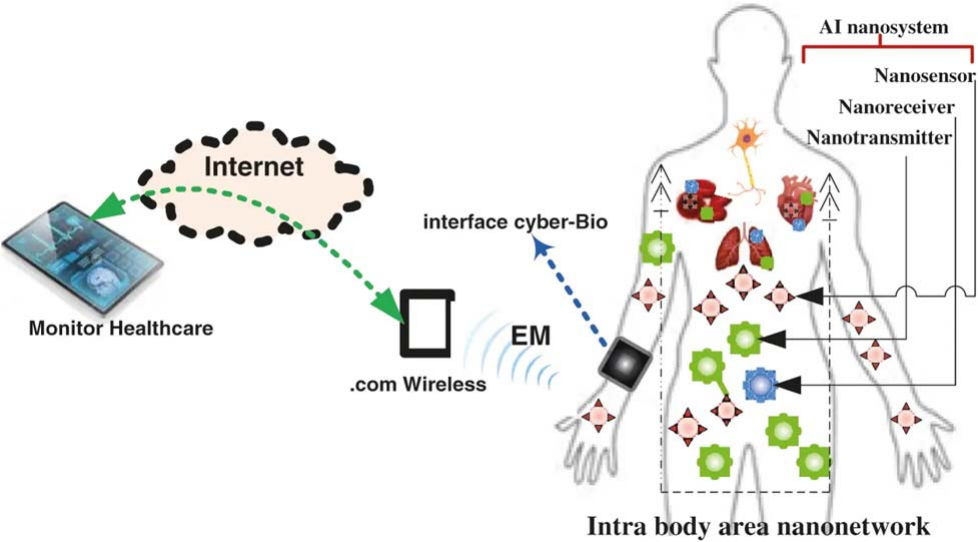
IoBNT-based AI nanosystem. Reprinted with permission from ref. 58. Copyright 2022 Tech Science Press.

Recent developments in nanotechnology have enabled researchers to create artificial nano-scale processes that closely mimic natural cell systems and study intra-body systems. There are numerous uses for this method, especially in the field of nanomedicine. The nanotransmitter nanomachine (TN) & the nanoreceiver nanomachine (RN) are the two nanoscale devices that make up a nanosystem. Systems for advanced targeted drug delivery (ATDD), illness detection and therapy, and healthcare monitoring are just a few medicinal uses for such an implanted device. Drug delivery to the intended location can be managed with the help of AI technology. Additionally, because of the coronavirus disease (COVID-19), medical professionals can stay healthy by using AI based on the Internet of Biological Nanothings (IoBNT) to communicate with their patients remotely.^53^ As IoT-based social networks rely on AT technology, Internet of Business Networks (IoBNT) is constructed on top of the IoT to facilitate communication and collaboration between AI nanosystems and external networks such as the Internet.^54,55^ On the other hand, the biocyber interface's function is to convert electromagnetic (EM) waves into biological signals.^56,57^ As seen in (Fig. 3), the following AI nano-scale gadgets are used to create the intra-body area network-based molecular communication (MC) system: TN for drug information molecule emission, RN for reception, and molecular channels for propagating (vascular blood medium).^58^ By elevating administrative tasks, medical research, diagnosis, and treatment planning, artificial intelligence has the potential to revolutionize healthcare.^59^ This review highlights different AI-based nanomaterials applied for biomedical applications.

## 5. Nanomaterial-based AI for biomedical applications

Due to their modern properties, nanomaterials work like magic in biomedical applications. Nanomaterials are widely used in the biomedical fields of antibacterial activity, diagnosis, scaffolds, culture of cells, regenerative medicine, drug transport, and tissue engineering.^50^ Due to improvements in characterization techniques and unique features, many nanomaterials, including MXenes, polymers, graphene, and CNTs, are now widely used in the biomedical industry as biosensors and bioimaging.^60–62^

**Fig. 4:**
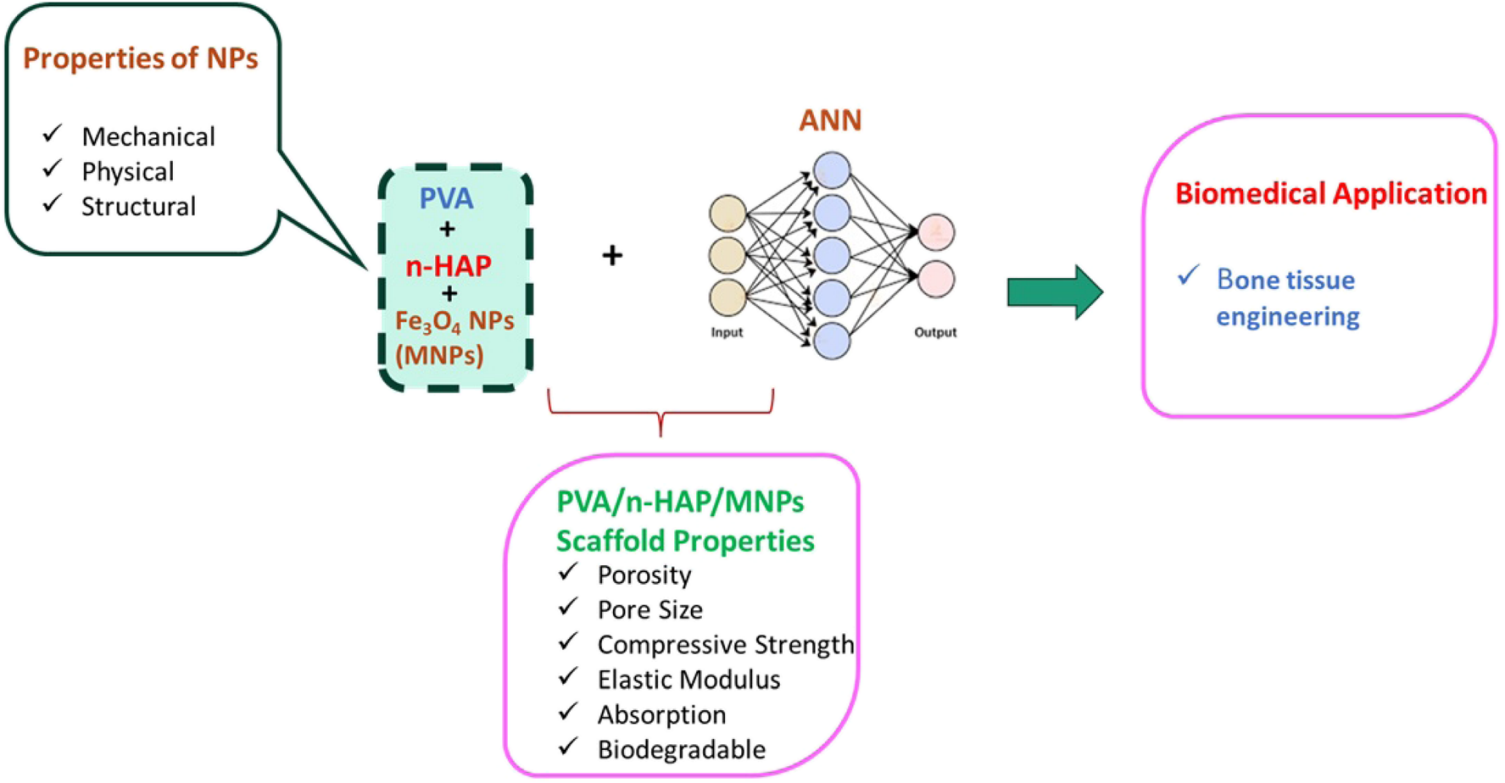
Schematic representation of the combination of nanomaterials with artificial intelligence (AI), especially artificial neural networks (ANNs), and its application.

Iron oxide (Fe_3_O_4_) nanoparticles (MNPs) and hydroxyapatite nanoparticles (n-HAP) were combined with polyvinyl alcohol (PVA) using a freeze-drying method to create a bioresorbable scaffold (Fig. 4a). PVA–n-HAP–MNPs (PHMs), biodegradable magnetic nanocomposite scaffolds, are used in bone tissue engineering. As the wt% of MNPs and n-HAP increased, the scaffolds' mechanical qualities improved in accordance with the ANN predictions. Furthermore, the scaffolds' porosity increased, and their pore size reduced. The structure and form of these scaffolds (Fig. 4b) were examined using SEM. The PHMs with the lowest weight wt% of n-HAP exhibited the thick, homogeneous, smooth, sheet-like structure produced by PVA. However, the scaffold's structure and shape changed, & smaller pore diameters were seen as further n-HAP and MNPs were added. Neural networks in deep learning, machine learning, and AI allow programs to recognize patterns and address common problems.^63–66^ ANN analysis offers convincing proof that adjustable control over scaffold features such as pore size and dynamics is possible by varying the nanoparticle content (Fig. 4). The PHM method's computational prediction and ability to enhance scaffold composition towards desired performance make it promising for the customized growth of bone regeneration materials.^67^

**Fig. 5:**
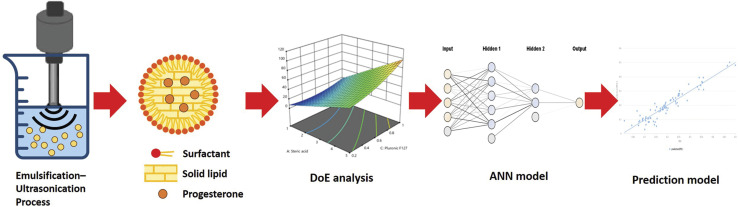
The generation of PG-SLNs using DoE and AI. Reprinted with permission from ref. 68. Copyright 2024 Elsevier.

The development of nanomedicine has the potential to be revolutionized by AI. Suriyaamporn *et al.* examined the physicochemical properties of progesterone-loaded solid-lipid nanoparticles (PG-SLNs) made by emulsification–ultrasonication, with an emphasis on using artificial neural networks (ANNs) and Design of Experiments (DoE) to show the effectiveness of this regulated preparation technique. The process of developing PG-SLNs utilizing AI and DoE is depicted in Fig. 5. For the first time, PGSLNs are being developed as a transdermal drug delivery method to help postmenopausal women delay the progression of neurodegenerative diseases. The scarcity of research projects in this field makes this study difficult to complete. This study aimed to create and develop PG-SLNs by utilizing AI and DoE approaches to forecast the best formulations for transdermal delivery of drugs in individuals suffering from Alzheimer's disease.^68^

One of the major challenges to practical applications of cancer nanomedicine is the insufficient ability of NPs to target solid tumors. To solve this conundrum, innovative machine learning and AI techniques and rapid processing capability expansion provide new tools. To assess the distribution in the body and tumor-targeted delivery efficiency (DE) of several NPs, Chou *et al.* combined a quantitative structure–activity relationship (QSAR) model with a PBPK model to create an AI-assisted physiologically based pharmacokinetic (PBPK) model. To predict significant input components of the PBPK model, the AI-based QSAR model was created using machine learning and deep neural network techniques. It had been trained utilizing data from a publicly accessible “Nano-Tumor Database”.^69^ A genetic condition affecting connective tissue called Marfan Syndrome (MFS) can cause a variety of phenotypes that can affect many different body systems, most notably the thoracic aorta. Distinct facial features are frequently present in the syndrome, which may facilitate clinical diagnosis and recognition. The Saksenberg research group investigates if face image analysis AI may be used to diagnose Marfan syndrome.^70^

**Fig. 6:**
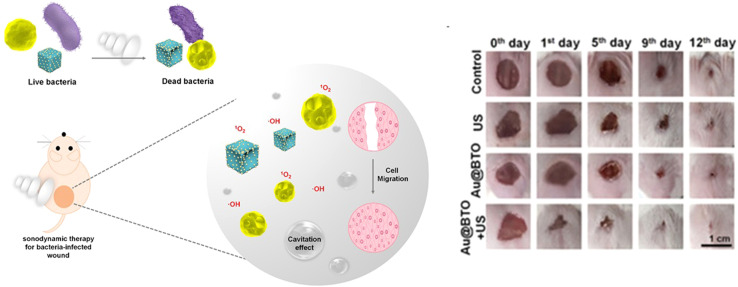
Au@BTO, a sonosensitizer for sonodynamic therapy. Reprinted with permission from ref. 71. Copyright 2021 Elsevier.

A novel form of biocompatible and highly effective sonosensitizer for highly effective sonodynamic therapy was recently introduced by Wu *et al.* as a piezoelectric nanocomposite known as barium titanate (BaTiO_3_, BTO) nano-cubes that has a modified Schottky junction using gold nanoparticles (Au@BTO), as illustrated in Fig. 6.^71^ A very accurate and least intrusive method of treating cancer and getting rid of infections is sonodynamic treatment.^72^

Using MXene-based gas sensors is an effective way to detect acetone, a biomarker of diabetes.^73^ Wang *et al.* used a chemiresistor of an α-Fe_2_O_3_/MXene heterostructure composite to create a sensor for the detection of acetone.^74^ Excellent acetone selectivity in the manufactured sensor makes it a useful instrument for early detection of diabetes and general health monitoring.^74,75^ Biosensors that include MXenes have shown great promise in identifying a range of illnesses in living things. A hybrid FET detector was created, beginning with Ti_2_C MXene/graphene, to monitor many viruses, including the influenza virus and SARS coronavirus-2. The biosensor showed that it could detect viruses between 25 and 250 000 copies per milliliter.^76^

The likelihood of a cure for cancer is increased with early detection. Techniques for cancer detection and clinical diagnosis that are accurate and dependable are urgently needed.^77^ Medical technological advancements have contributed to a decrease in the death rate from cancer; nevertheless, patient survival can still be improved and fatalities can still be reduced with early diagnosis, regular monitoring, and the use of multiple PoC devices. Wearable sensors, the IoT, AI, lab on a chip, organ on a chip, and next-generation sequencing (NGS), which evaluate a person's physical and psychological biometrics, can be used to provide a unique and technological strategy for cancer control.^78^

An increasing number of recent studies have shown how very effective GO and its components are in the biosensing of cancer and in diagnostics (theragnostic).^79^ AI technologies, such as neural networks, are essential tools for the diagnosis of cancer. Recently, researchers created an AI-based discriminating system for different gas assessments and a breath analysis sensor array employing multiplexed DNA-functionalized graphene nanoelectrodes. By examining an individual's EB, this sensor array can be utilized to diagnose illnesses early on. Its capacity to differentiate between different mixed chemical gas compositions has also been verified. A study used 1D convolutional neural networks (CNNs) to achieve a 98% accuracy rate in gas classification.^80^ The newly developed chemo-resistive sensors aim to detect biomarkers for lung cancer in artificial breath samples using many indicators and have a robust accuracy of 96.5%.

**Fig. 7:**
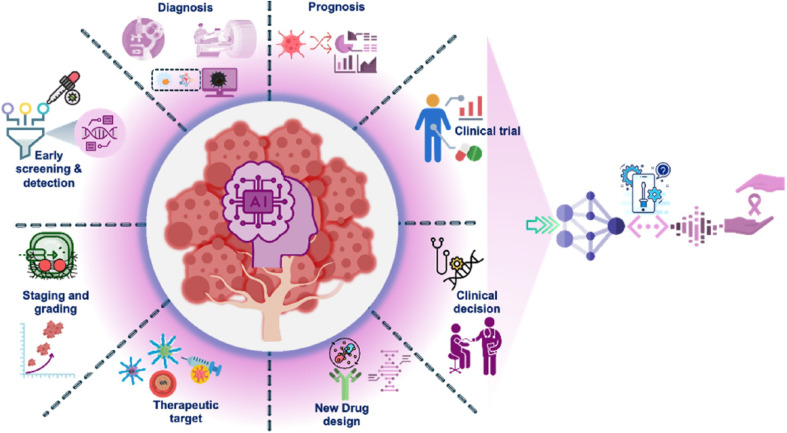
An innovative representation of graphene combined with AI and IoT. Reprinted with permission from ref. 82. Copyright 2024 John Wiley & Sons Australia.

Since graphene can be used to make wearable electronics that simulate human sensations, including taste, smell, touch, and hearing, it has drawn attention to AI. Using machine learning techniques, these sensors identify tasks and determine the external environment while offering output signals.^81^ Combining machine learning techniques with graphene oxide (GO)-based devices enhances cancer therapy.^82^ The challenges and opportunities associated with combining ML, AI, and GO-based sensors for cancer treatment are given in Fig. 7. The implementation of sensor technology is addressed with its challenges and obstacles.

Due to its high sensitivity, affordability, and convenience, breath testing for carbonyl cancer markers may someday be utilized as the main technique for screening for lung cancer.^83^ Graphene may be used in VOC sensors that detect cancer *via* breath analyzer emissions because of its sensitivity to volatile organic compounds (VOCs).^84^ GO produced by metal ions is used by Chen *et al.* as an e-nose for the breath-exhalation approach to diagnosing lung cancer.^85^ Recently, researchers created an AI-based discriminating system for different gas assessments and a breath analysis sensor array employing multiplexed DNA-functionalized graphene nanoelectrodes. By examining an individual's EB, this sensor array can be utilized to diagnose illnesses early on. Its capacity to differentiate between different mixed chemical gas compositions has also been verified. A study used 1D convolutional neural networks (CNNs) to achieve a 98% accuracy rate in gas classification.^86^

AI-powered nanobots, tiny robotic robots capable of doing challenging tasks at the microscopic level, result from merging these technologies. The advantages that AI-powered nanobots can offer people in early disease detection, intervention, and surgery are examined in this research. Together, AI and nanotechnology provide highly precise and efficient early disease detection. These nanobots can directly target damaged cells or tissues using their small size and advanced capabilities, reducing side effects and improving patient healing efficacy. AI-capable nanobots can significantly enhance surgical techniques. They can assist surgeons in performing delicate and complex surgery by providing real-time feedback, increasing accuracy, and lowering risks.^87^ Nanotechnology is a promising area for transforming the early detection and surgical treatment of stomach tumors. Drug inefficiency and high rates of recurrence from surgical and pharmaceutical therapy are the main factors affecting curative efficacy in patients with GIC (Gastrointestinal Cancer). Given the drawbacks of GIC MRI and endoscopy and the difficulty of gastric surgery, nanotechnology has shown promise in the early identification and timely treatment of stomach issues. By specifically targeting cancerous cells, nanoparticles can identify and destroy them. To improve the accuracy and effectiveness of cancer treatment, it can also be designed to deliver particular payloads, including medications or contrast chemicals. By employing XGBoost and RNN-CNN as a classification method, the current study used the improving machine learning method to find nonlinear interactions among many variable's inputs and outputs. With impressive metric values showing the promise of cutting-edge machine learning and nanotechnology for the diagnosis and treatment of gastric cancer (GIC), the study's findings are quite optimistic. In the examination of stomach images, the model's outstanding accuracy (93.54%), precision (93.65%), *F*_1_-score (93.57%) & recall (93.87%) were followed by an excellent area under the curve of 93.54%, indicating its great discriminatory ability. These results show how effective the integrated approach is and suggest that it might significantly improve GIC evaluation and therapy, which would ultimately give hope for more precise, tailored, and successful results in the battle against this challenging condition.^88^

As a commonly used anticoagulant with several clinical side effects, heparin necessitates stringent quantitative and qualitative screening to protect patients from the risk of thrombocytopenia. However, the low availability and complexity of heparin's chemical structures make it difficult to precisely monitor its level and quality in therapeutic settings.

Due to the sensitivity and specificity limitations of conventional laboratory assays, novel strategies must be developed. Because of their improved sensitivity, selectivity, and capacity for detecting heparin even at low concentrations, nanosensors have emerged as a possible solution in this regard. Nanosensors could be incorporated into wearable technology, such as wristbands or smartwatches, to continuously check the amount of heparin in the user's blood. Additionally, these devices may be incorporated with AI/machine learning (MI) algorithms which may evaluate huge data sets to assist with the selection of particular sensor characteristics, such as materials or operating parameters, as demonstrated in point-of-care biomarker detection for diseases such as cancer, diabetes, and coronavirus disease (COVID-19).^89–94^ This method makes use of AI/ML's skills in pattern recognition, data processing, and sensor design optimization.

Comparable to the human bionic approach, connecting the human nervous system to computers allows for previously unheard-of control over prosthetic limbs and the restoration of lost sensory function. Such a cutting-edge technical approach represents an important step toward improving ANNs, which are essential for understanding how AI works, how to create Super Artificial Intelligence (SAI), and how crucial nanoscience and nanotechnology are to this cutting-edge technology, which also determines a drug delivery system based on nanotechnology.^95^ We now fast-forward forty years. During the 2014 World Cup in Brazil, neuroprosthetics attracted significant public interest once more. A neuroprosthetic gadget created by Dr Miguel Nicolelis' group at Duke University enabled a patient with spinal cord injuries to begin the World Cup. The development of the latest version of AI, which we now refer to as super artificial intelligence (SAI), is greatly advanced by this.^38^

Combining nanotechnology and AI in medicine improves precision cancer treatment. Two areas that are crucial to achieving precision medicine's objective of customizing the optimal course of treatment for every cancer patient are AI and nanotechnology. Machine learning and AI have the potential to create “smart” nanoengineered Brain–Machine Interfaces (BMIs) due to a convergence of technological capabilities. Communication will be possible with this new generation of technology, with the brain working in ways that facilitate contextual learning and flexibility in response to evolving functional needs. This is true for both noninvasive technologies made possible by signals such as the electroencephalograph (EEG) and intrusive technologies such as the neural prosthesis that are intended to restore neurological function.^95^

## 6. Conclusion and future perspectives

AI has the potential to revolutionize patient care, improve public health surveillance, optimize healthcare operations, personalize treatment programs, and increase diagnostic accuracy. The dynamics of human-AI interaction, data privacy and security, legal and moral concerns, interoperability and integration problems, scalability and accessibility issues, and other challenges must all be navigated in order to fully realize the potential of AI in healthcare. While working effectively, AI is more advantageous; yet, it cannot take the place of the interpersonal relationships that build teams. It is impossible for robots to do human activities such as cooperation and team management because they are unable to develop emotional connections with people. Verifying that AI can be developed and applied in a way that respects people's interests and takes into consideration technological, ethical, and social factors will be a major problem for the regulation of AI technologies in the future.

Combining AI with nanotechnology represents a fundamental paradigm shift with wide-ranging implications for businesses and scientific domains. AI-driven nanotechnology is revolutionizing healthcare and illness management by accelerating the creation of customized drugs and targeted therapies. The development of tailored properties for a variety of applications, such as electronics and aerospace, is made easier by AI-driven breakthroughs in nanotechnology, which benefits materials science and engineering.

## Data availability

No primary research results have been included and no new data were generated or analysed as part of this review.

## Author contributions

Subhendu Chakroborty: supervision, conceptualization, writing – review & editing. Nibedita Nath: writing – original draft preparation, editing. Sameeta Sahoo: writing – review & editing, investigation. Bhanu Pratap Singh: investigation, formal analysis. Trishna Bal: conceptualization, writing – review & editing, investigation. Karunesh Tiwari: supervision, visualization, writing – review & editing. Yosief Kasshun Hailu: data curation. Sunita Singh: writing – original draft. Pravin Kumar: writing – original draft, formal analysis. Chandra Chakraborty: resources, methodology.

## Conflicts of interest

There are no conflicts to declare.
